# Desires and Needs for Quitting Both e-Cigarettes and Cigarettes Among Young Adults: Formative Qualitative Study Informing the Development of a Smartphone Intervention for Dual Tobacco Cessation

**DOI:** 10.2196/63156

**Published:** 2024-10-22

**Authors:** Nhung Nguyen, Kimberly A Koester, Christine Tran, Pamela M Ling

**Affiliations:** 1 Center for Tobacco Control Research and Education University of California, San Francisco San Francisco, CA United States; 2 HEARTY lab University of California, San Francisco San Franscisco, CA United States; 3 Division of General Internal Medicine Department of Medicine University of California, San Francisco San Francisco, CA United States; 4 Center for AIDS Prevention Studies Division of Prevention Science University of California, San Francisco San Francisco, CA United States

**Keywords:** smoking cessation, vaping cessation, mHealth intervention, mobile health, e-cigarettes, cigarettes, smartphone intervention, nicotine dependence, additive adverse health effects, tobacco cessation

## Abstract

**Background:**

Dual use of both e-cigarettes and cigarettes is popular among young adults and may lead to greater nicotine dependence and additive adverse health effects than single-product use. However, existing cessation programs target quitting either e-cigarettes or cigarettes, highlighting a need for interventions to help young adults quit both products (ie, dual tobacco cessation).

**Objective:**

This formative study is part of a larger project to develop a smartphone intervention for dual tobacco cessation among young adults. This study aimed to (1) explore desires for and experiences with quitting both e-cigarettes and cigarettes and (2) identify needs and preferences for dual tobacco cessation intervention programming.

**Methods:**

Semistructured interviews were conducted to elicit the need for and experience with dual tobacco cessation among 14 young adults (18-29 years old) recruited through Instagram (Meta) advertisements in 2023. We conducted a thematic analysis to identify common themes related to quitting experiences and cessation needs.

**Results:**

Participants expressed a strong desire for dual tobacco cessation and had attempted to quit both tobacco products, mostly “cold turkey.” The priority product for quitting first varied by the individual’s perceived harm or level of consumption. Targets for dual tobacco cessation interventions included (1) highlighting the health effects of dual tobacco use compared with single product use, (2) providing cessation support to quit one prioritized product while cutting down the other product with the explicit goal to quit both, (3) emphasizing unique facilitators and barriers to quitting each product (eg, unpleasant smell of cigarettes facilitating smoking cessation and accessibility and flavors of e-cigarettes hindering vaping cessation), and (4) addressing co-use of tobacco with alcohol or cannabis. Participants wanted personalized interventions through smartphone apps that would tailor support to their tobacco use patterns and unique quitting goals and needs. They also suggested presenting intervention content in multimedia (eg, videos, graphic pictures, quizzes, and games) to increase engagement.

**Conclusions:**

This study provides important insights into young adults’ experiences, needs, and preferences for dual tobacco product cessation. We highlight important targets for future smartphone apps to deliver personalized and tailored support to meet the heterogeneous needs and preferences of young people who want to quit using both e-cigarettes and cigarettes.

## Introduction

Young adulthood (18-29 years old) is an ideal time for tobacco cessation to prevent the escalation of tobacco use and related diseases [1]. Tobacco cessation among young adults is complicated by concurrent use of both e-cigarettes and cigarettes (dual use) [2]. Among young adults who currently used e-cigarettes in 2021, 37% also reported using cigarettes [3]. Dual use may lead to greater nicotine dependence and additive adverse health effects than single-product use [4]. Thus, quitting both products (ie, “dual tobacco cessation”) should be a goal to prevent health consequences. However, most cessation programs target either e-cigarettes or cigarettes, highlighting a need for interventions addressing dual tobacco cessation for young adults [5].

Evidence-based treatment for tobacco cessation includes a combination of pharmacologic and behavioral support [6,7]. However, evidence is limited on which strategies are particularly effective for young adult dual tobacco cessation [8]. Compared with those exclusively using e-cigarettes, young adults who use both cigarettes and e-cigarettes reported higher importance but lower confidence in quitting all tobacco and nicotine products [9]. Given differences in use and cessation characteristics, strategies effective for quitting a single product may not work for dual tobacco cessation [10]. It also needs to be clarified if young adults prefer to quit e-cigarettes and cigarettes simultaneously or sequentially. Thus, understanding young adults’ quitting experiences and cessation needs is critical to guide the development of dual tobacco cessation interventions.

Previous reviews have highlighted smartphone apps as a promising channel to reach and deliver tobacco cessation support to young adults, given the widespread use of smartphones in this age group [5,11]. A 2023 review of smartphone apps for youth tobacco use prevention and cessation found 4 interventions targeting cigarette smoking and 1 targeting nicotine vaping [12]. These apps, however, were in the early stages (eg, formative research and pilot or feasibility testing) with limited evidence on efficacy or effectiveness for tobacco cessation. Another review identified 8 apps available in Canada (3 specifically targeting vaping cessation and 5 focusing on smoking cessation while also claiming to address vaping cessation) and found that these apps had limited features explicitly developed for vaping cessation and had little evidence-based content [13]. Likewise, a 2024 systematic search found 6 apps available in Australia designed for vaping cessation; however, these apps lacked trials or testing [14]. These reviews further highlight the need for future apps to consider the actual needs of target users rather than simply adding vaping cessation content to existing smoking cessation apps. To this end, this formative study is part of a larger project to develop a smartphone-based intervention for dual tobacco cessation among young adults. As a first step, this study aimed to gain insights into young adults’ perspectives on dual tobacco cessation. Our research aims were to (1) explore desires for and experiences with quitting both e-cigarettes and cigarettes and (2) identify needs and preferences for dual tobacco cessation intervention programming.

## Methods

### Design and Participants

We conducted semistructured interviews with 14 young adults from January to April 2023. Participants were recruited through Instagram (Meta) advertisements linked to study information and eligibility screening. Inclusion criteria were (1) aged 18-29 years old, (2) using both cigarettes and e-cigarettes in the past 30 days, (3) motivated to quit either tobacco product in the next 6 months, (4) owning a smartphone, and (5) living in California. Eligible participants were asked to provide a photo ID for verification. The participants resided in different cities or areas in California. Initially, participants were asked to complete an online Qualtrics survey with tobacco use history and demographic characteristics. They were then invited to a Zoom (Zoom Video Communications) interview.

### Data Collection

Trained interviewers (NN, KAK, and CT) with various experiences in tobacco control research conducted 60-minute interviews with participants. We developed the interview guide based on the literature about young adults’ tobacco use and cessation [7,15,16]. Key constructs included desire and past attempts at quitting, facilitators and barriers to quitting tobacco, preferences for cessation supports, and suggestions for interventions (refer to Multimedia Appendix 1 for complete interview guide).

### Data Analysis

Participants’ demographic information and tobacco use characteristics reported in the Qualtrics survey were summarized using descriptive statistics. All interviews were audio recorded, transcribed verbatim, and reviewed for accuracy. Thematic analysis was conducted on the Dedoose (SocioCultural Research Consultants) platform following 6 phases, such as familiarization with data, generating initial codes, searching for themes, reviewing themes, defining and naming themes, and producing the final analyses [17]. NN read all the transcripts to achieve immersion in the data and developed a coding scheme, including a priori or deductive codes based on the interview guide and new or inductive codes that emerged during the analysis. In addition, 2 research assistants (CT and Sara Perez) independently applied codes to all the transcripts. Coding disagreements were discussed and resolved by the entire team. We grouped coded data into potential themes, identified main themes, and interpreted results. We reported results following the Standards for Reporting Qualitative Research guideline [18] (refer to Multimedia Appendix 2 for the reporting guideline).

### Ethical Considerations

The University of California, San Francisco’s Institutional Review Board approved the study (IRB 22-37048). All participants signed informed consent forms to take part in the study and were informed of their right to withdraw from the study at any time without any consequences. An identification number was assigned to each participant to maintain anonymity, and all data were deidentified. Participants received a US $100 e-gift card for their participation.

## Results

### Sample Characteristics

Participants’ mean age was 23 (SD 2.9) years, with the majority being male (8/14, 57%) and heterosexual (8/14, 57%); 50% (7/14) were non-Hispanic Asian, and 43% (6/14) were currently in college (Table 1). Many participants had tried to quit either e-cigarettes (12/14, 86%) or cigarettes (10/14, 83%) in the past 12 months, 71% (10/14) intended to quit e-cigarettes, and 43% (6/14) intended to quit cigarettes in the next 6 months.

**Table 1:** Sample characteristics (N=14).

Demographic characteristics				Value, n (%)
**Age (years), mean (SD)**				23.0 (2.9)
**Sex at birth**				
	Male			8 (57)
	Female			6 (43)
**Sexual identity**				
	Heterosexual			8 (57)
	LGBTQ+^a^			5 (36)
	Decline to state			1 (7)
**Education**				
	Currently in college			6 (43)
	Currently in graduate school			2 (14)
	Currently in professional or technical school			2 (14)
	Not attending school			4 (29)
**Race and ethnicity**				
	Non-Hispanic White			1 (7)
	Non-Hispanic Black			3 (21)
	Non-Hispanic Asian American Pacific Islander			7 (50)
	Hispanic or Latino/a/x			3 (21)
**E-cigarette use characteristics**				
	Number of days using e-cigarettes in the past 30 days, mean (SD)			21.9 (8.0)
	Number of times per day using e-cigarettes, mean (SD)			5.4 (4.7)
	Have tried to quit using e-cigarettes in the past 12 months, n (%)			12 (86)
	**Intention to quit using e-cigarettes, n (%)**			
		In the next 30 days	4 (29)	
		In the next 6 months	10 (71)	
**Cigarette use characteristics**				
	Number of days smoking cigarettes in the past 30 days, mean (SD)			10.5 (10.8)
	Average number of cigarettes smoked per day, mean (SD)			3.5 (3.7)
	Have tried to quit smoking in the past 12 months, n (%)			10 (83)
	**Intention to quit smoking, n (%)**			
		In the next 30 days	5 (36)	
		In the next 6 months	6 (43)	
		No intent to quit in the next 6 months or missing	3 (21)	

^a^LGBTQ+: lesbian, gay, bisexual, transgender, and queer or questioning.

### Desire for Dual Tobacco Cessation

Of the 14 participants, 2 intended to quit both products in the next 30 days, and 6 intended to quit both products in the next 6 months. When asked about their desire to quit smoking and vaping, there was a heterogeneity of preferences for the timing and order of products to quit. Some participants conveyed a willingness to quit both tobacco products simultaneously because it set “a very clear boundary” for not using nicotine or tobacco:

I’m kind of like all in or nothing. So, it’s easier for me to just get rid of everything [all tobacco/nicotine products] I have and commit to not doing it than to wean myself off.

R08, 25-year-old Hispanic female participant

Another noted that dual tobacco cessation could prevent switching or increasing the use of either product:

I probably should quit both of them. The thing is, if I stop vaping, I’ll crave cigarettes more.

R04, 22-year-old Hispanic male participant

Other participants described a desire to quit 2 products sequentially. When being asked about the reasons why quitting one product (eg, cigarettes or e-cigarettes) was more important than quitting the other product, those who wanted to quit cigarettes first perceived that quitting smoking was more important due to cigarettes’ higher costs and adverse health effects:

For smoking, I feel that needs to kind of go away, especially since I know all the side effects that come along with it. So, smoking is more of something I’m trying to get away from. [Quitting] vaping is not as high up. I would say because it’s [quitting vaping] just easier. Out of all the things that I could be worrying about, not doing that one isn’t at the top of the list so it’s not what I go for at first.

R02, 22-year-old Asian male participant

Conversely, other participants prioritized quitting vaping because they used e-cigarettes much more frequently than cigarettes:

I smoke cigarettes only on occasion, I don’t think they’re as harmful for me. If it’s like occasionally, like once or twice a month, then it’s okay. But, when it becomes a daily habit, like vaping, I think that’s the most important thing I need to focus on stopping.

R04, 22-year-old Hispanic male participant

### Strategies Used and Not Used During Quit Attempts

When being asked about whether they had tried to quit in the past and what types of support they had used for quitting, most of the participants reported having attempted to quit either or both tobacco products, most frequently “cold turkey”:

A lot of people quit cold turkey because they don’t know how else to do it. They don’t know where to go to for support. They don’t have any ideas. They don’t have any plans. So, there’s kind of like the lack of support.

R03, 26-year-old Asian female participant

Participants described various personal strategies to deal with nicotine cravings (eg, using non-nicotine e-cigarettes, drinking water or coffee, chewing mint gum or sunflower seeds, eating snacks or chocolate, exercising or meditating, and biting fingernails). Notably, several participants used cannabis to quit e-cigarettes and cigarettes because cannabis products had similar routes of administration to tobacco products:

When I was quitting, I would crave the oral fixation of smoking. Well then, I guess I’ll smoke weed, completely different situations, but you’re still smoking something. And they have cannabis pens too. So, when I first quit vaping, I just bought - there’s a brand called STIIIZY. And STIIIZY has a very similar build to a vape pen of tobacco product. I feel like it was easy for me to just hold the STIIIZY and feel like I had my vape and that I could hit it at any point.

R08, 25-year-old Hispanic female participant

None reported using medication (eg, nicotine replacement therapy [NRT]) since they perceived NRT as a “wake-up call” for one’s addiction or loss of control over tobacco usage. Young adults considered their tobacco use akin to a recreational hobby or casual activity rather than an addiction. Interestingly, 1 participant thought of NRT as just another form of nicotine that would not help with cessation:

I don’t want to keep consuming more nicotine in a different form. That [NRT] doesn’t seem like it would be helpful for me. It just seems like still using the same substance that I know is being harmful to my body...I don’t think that switching the form of how you’re consuming the substance is helpful to quitting because it’s still giving your body what it wants.

R14, 23-year-old Hispanic female participant

Furthermore, participants avoided asking for help from health care providers as it was embarrassing to disclose their tobacco use:

Actually, first, I was considering going to my school counselor because we have free counseling. But the thing is, because it’s a Christian institution, and I don’t know if the counselor would say something to the school because it’s technically like, well, you’re not supposed to [use tobacco]...I can’t use that method because I don’t really want to give them sensitive information that goes against university values.

R12, 20-year-old Asian male participant

### Barriers to Quitting or Intervention Opportunities

When asked about barriers to quitting tobacco, participants described similarities and differences between quitting smoking and quitting vaping. Common barriers to quitting both tobacco products included habitual use, stressors, withdrawal symptoms, social settings, and triggers by cannabis or alcohol.

Many participants found it hard to quit because tobacco use had become a daily habit:

Sometimes, I use cigarettes. But, most of the time, it’s vaping. I probably go through about like a pod every two to three days. Vaping is more like an everyday thing...I think now it’s just such a habit and I feel like, when I do have the urge and I’m like not doing anything else or like I’m alone or like I’m at home or outside, I feel like it’s just kind of like why not [vaping] if I already have it [e-cigarette] and it’s like right there.

R10, 23-year-old Asian female participant

Participants also mentioned common transitional stressors during young adulthood that made quitting difficult:

I used smoking as a copout in some ways, where I was like, “This really stressful thing just happened to me. I’m going to smoke to make myself feel better about it.” And recently, with the recession and with trying to transition into a full-time career post-COVID has been really difficult [to quit tobacco].

R09, 23-year-old Asian female participant

Participants were often surrounded by people who use tobacco, making social settings “not going to be the most encouraging environment” to quit e-cigarettes and cigarettes. Another barrier to quitting was withdrawal symptoms (eg, irritation, nicotine craving, lack of focus, insomnia or troubling sleep, headache, bad mood, anxiety, restlessness, and dehydration):

I feel like the withdrawal period for nicotine lasts at least several months. That is a long time of being irritable, of being stressed, of being irritated, like not a sense of calmness. So, I can’t afford to take that withdrawal period, because life is so busy.

R03, 26-year-old Asian female participant

Alcohol and cannabis were triggers for tobacco use due to overlapping effects between nicotine and these substances, as 1 participant elaborated:

Whether it’s if you’re smoking marijuana or you’re drinking, that is a big trigger that makes me want to vape more in those moments when I start to feel those [overlapping] effects.

R14, 26-year-old Hispanic female participant

In addition to the common barriers, unique barriers to quitting vaping were appealing flavors and easy accessibility or convenient use of e-cigarettes. R09 described e-cigarettes as “a half-and-half” of nicotine and flavors:

With a cigarette, I’m smoking it mostly for the nicotine. Whereas, a vape - it’s such a good balance of the flavor and being able to hit it whenever I want but also having the nicotine that will calm me down.

R09, 23-year-old Asian female participant

### Needs and Preferences for Dual Tobacco Cessation

#### Combined or Separate Interventions

When asked whether smoking cessation and vaping cession should be combined or separated, participants expressed a variety of individual needs and preferences for dual tobacco cessation interventions without a clear preference for a combined intervention or separate interventions. Some preferred one intervention to “stop consuming nicotine in any form” as there were many similarities between cigarettes and e-cigarettes.

If there’s some plan or something on how to stop smoking, then probably it could also apply to vaping, too, because they’re pretty much the same.

R01, 21-year-old Asian male participant

Another participant also supported using one intervention for dual tobacco cessation because:

...*if someone uses an app and puts in what they’re trying to quit, then maybe the app can tailor it based on if it’s smoking or vaping. So, as long as an app takes that into account, I think it could do both.* [R04, 22-year-old Hispanic male participant]

Conversely, others preferred to have separate interventions for each product because these products have different use characteristics (eg, time commitment and permitted location for smoking, smell of cigarettes, greater frequency and flavors of e-cigarettes).

Even though they do seem very similar, like apples and oranges, with vaping, I think the real appeal of it is the fact that it doesn’t smell and that it’s very easy to do, whereas with smoking it’s pretty socially unacceptable to smoke indoors...So, it would have to be a different approach.

R02, 22-year-old Asian male participant

Another participant even thought that more support would be needed for vaping cessation because:

...the vaping community is a whole different type of community than the cigarette people. When you’re vaping, you’re vaping at a higher frequency and a higher amount of nicotine. While, with cigarettes, it is a time and a place to do it appropriately, outside. With vaping, I feel like it needs more extra support of care because of how often you can do it anywhere.

R11, 23-year-old Asian female participant

R11 also noted another reason for separating interventions was limited data about the health impacts of e-cigarettes:

If you want people to stop [vaping], you want them to get more educated on something that they don’t know about. So, you have to offer them additional information on vaping because there’s not much data that is already created for it, while tobacco [cigarettes] has a sufficient amount of articles and data that is pretty accessible to the public.

R11, 23-year-old Asian female participant

#### Intervention Content

Participants suggested intervention content for a smartphone app, including concrete quit plans, frequent reminders of quitting benefits (eg, health benefits or money savings), and trackers of quitting progress (eg, number of days they stay free from vaping and smoking), alternative strategies for withdrawal management and anxiety or depression, peer or social support, and addressing tobacco product switching or co-use with alcohol or cannabis. For example, R11 suggested:

There should be like a button that talks about the long-term health effects of either tobacco smoking or vaping. And it should also probably talk about the common ingredients that are in vaping or in tobacco [cigarettes]. So, it has the user be educated on what chemicals they’re like putting in their body...There can also be another application where you can record your tobacco or vaping purchases. It can show you an index of how much it would be over X amount of years or months. So, you can be able to see how vaping is taking a toll on you, financially, or smoking tobacco has taken a toll on you, financially.

R11, 23-year-old Asian female participant

To address the need for mental health support, participants suggested offering mindfulness practice or breathing techniques since it could help them feel relaxed and reach the same feeling as “the head high from the nicotine.” R14 further recommended referrals to mental health services:

When people are depressed, they smoke more and vape more...So, if that’s the case, I’m thinking about the mobile application, a banner that pops up that says, “Are you struggling with fatigue or depression? Click here to find out for help.” And then that can lead to some sort of database that can help you find either telehealth or providers in your area.

R14, 23-year-old Hispanic female participant

In order to achieve quitting goals, participants also suggested creating “a sobriety tracker” in which people could see milestones in their quitting journey or using a leaderboard to compare their progress with other people who are also trying to quit. Interestingly, R12 suggested using sensor-based detection to track e-cigarette consumption automatically:

I feel like the app would be able to automatically calculate the puffs, like noise sensing. Fitbits and other fitness bands and everything have an edge of monitoring your heart or something like that.

R12, 20-year-old, Asian male participant

Some participants wanted to receive support from friends or to have their friends quit smoking and vaping together since it would create a supportive environment for quitting and reduce their accessibility to tobacco products. Relatedly, some participants preferred to have social support by learning about quitting experiences from others through a discussion board or a forum:

Maybe people could share their stories of quitting, like experience of quitting attempts...You could put like, you’re not alone. There’s a lot of people trying to quit, and most people are feeling the same withdrawal symptoms and everything. And there’s a lot of people who successfully quit vaping and cigarettes, and never give up...You could ask people what their coping ideas are, when they’re stressed out, what do they do when they get the urge. Tips and tricks.

R13, 23-year-old White male participant

Several participants mentioned the need to discourage the switch between e-cigarettes and cigarettes. R14 suggested that:

You could ask [people who try to quit vaping], “Have you noticed that you’ve been reaching for cigarettes more lately?” Or, if they’re mostly trying to quit cigarettes, “Have you noticed that you’ve been reaching more for e-cigarettes lately?”...And then you can do something with that. Maybe a link to an article that discusses these patterns [switching between e-cigarettes and cigarettes] and what they could possibly do to curb that.

R14, 23-year-old Hispanic female participant

This participant also suggested addressing co-use of tobacco with cannabis or alcohol:

I think you could send a question, maybe around the evening when people typically do those things. And you could be like...Are you drinking or smoking a weed? If you are, remember to put your vape away for the night while you do it.

R14, 23-year-old Hispanic female participant

#### Intervention Format

All participants strongly supported an app-based intervention for cessation support as R01 said:

I’m always on my phone. Everything you can track through your phone. So, if you have an app that you could use to cut [reduce/quit tobacco use], to put down what’s your goal or something, that would actually be good. Or it tells you what you should do or sort of instructions. That would be great. I can do it on my own time and pace.

R01, 21-year-old Asian male participant

R14 (23-year-old Hispanic female participant) further highlighted that app interventions could help young adults quit tobacco use anonymously and avoid related stigma when seeking support from others.

Participants suggested presenting intervention messages in graphic pictures, quizzes, and games to increase young adults’ engagement with an app since these formats would be more fun and engaging than SMS text messages alone. Interestingly, R08 (25-year-old Hispanic female participant) wanted to have podcasts about motivational messages or quitting tips so that she could listen while driving. Some participants suggested having video-based content featuring quitting advice from relatable young people, as R03 said:

Young people are addicted to TikTok as much as they are to vaping nicotine...So, I think maybe portraying it in a video would be really helpful. Because if someone sees that someone’s already doing [quitting], they’re like, “Okay, I can relate to that. Like, I can do that too.”...That would make a more lasting impression...But if a grown person, a doctor, a medical professional is being like, “Oh, this is some advice,” it’s harder to make an impact on you. Because you don’t feel a relatability to them. This is a doctor. He’s not addicted to vaping.

R03, 26-year-old Asian female participant

#### Personalized Supports

All participants recommended interventions that could be customized to meet their individual needs, including “curating your own personal treatments” based on their tobacco use patterns and current stage in the quitting journey. For example, R12 said:

“*If the app had something that could split maybe into categories, like one category would be severe vapers - someone who really wants to quit. Another category could be someone who has quit but they’re struggling and craving and want to do it again...Also, it could categorize based on your experiences...It becomes more personalized. You feel like it’s really catered toward you, like, this category was created for me...Because I feel like everyone has their own personal custom schedule. So, I feel like it didn’t really make any sense to me, like a generalized schedule to quit.”* [R12, 20-year-old Asian male participant]

## Discussion

### Principal Findings

This study provides important insights into young adults’ experiences, needs, and preferences for dual tobacco cessation. Young adults expressed a strong desire to quit both e-cigarettes and cigarettes, with 8 (57%) out of 14 participants intending to quit both products in the next 6 months, although we did not find a consensus on whether to quit both products simultaneously or sequentially. We found participants had previously attempted to quit either or both products unsuccessfully and mostly quit without plans or support. They were generally unwilling to use pharmacotherapy and had tried non–evidence-based strategies, including using cannabis products to quit tobacco products. A scoping review on dual tobacco cessation recommended supporting individuals to cease dual use first by providing initial support for smoking cessation followed by support for quitting vaping [19]. However, our study indicated that this approach may not work for all young adult dual users. In our sample, the priority product preferred to target for cessation varied across individuals. Participants prioritized the products perceived as producing greater harm or products with higher consumption levels for cessation.

The heterogeneous needs and preferences for dual tobacco cessation among our participants indicate the promise of personalized interventions that could tailor support to individuals’ tobacco use patterns, quitting goals, and unique needs, facilitating successful cessation. Although our sample included young adults who reported dual tobacco use, the average frequency and intensity of e-cigarette vaping (21.9, SD 8.0 days in the past 30 days and 5.4, SD 4.7 times per day) was greater than those of cigarette smoking (10.5, SD 10.8 days in the past 30 days and 3.5, SD 3.7 cigarettes per day). The predominant use of e-cigarettes in our sample may reflect the fact that e-cigarettes are the most commonly used tobacco products among young adults [20]. In addition, young people reported the use of e-cigarettes in times and places where smoking is not allowed or not acceptable, which may lead to increased frequency of vaping [2]. Previous research has shown that dual users are a diverse group with a variety of use patterns (ie, daily use of both cigarettes and e-cigarettes, predominant use of e-cigarettes, predominant use of cigarettes, or nondaily use of both products) and differing levels of nicotine dependence [21]. The variation in the quantity of smoking and vaping among dual users reinforces the need for personalized interventions. For example, more messages about vaping cessation may be needed for those who predominantly use e-cigarettes, while more messages about smoking cessation may be needed for those who predominantly use cigarettes. The need for personalized interventions was further reinforced as there was no clear consensus about whether to combine or separate interventions for dual tobacco cessation, with some participants preferring 1 intervention for quitting both tobacco products while others wanted 2 separate interventions. To make the intervention engaging, participants recommended using multimedia forms rather than SMS text–only messages. They were excited about using a smartphone app to receive the intervention so that they could access support anonymously at their own pace and time and avoid the stigma associated with tobacco use.

### Comparison With Previous Work

Our findings are consistent with the extant literature supporting the goal to quit all tobacco and nicotine products, most young adults quitting without evidence-based treatment support, and shared barriers and facilitators to quitting e-cigarettes and cigarettes [22]. Aligned with a previous qualitative study [16], we found that the greater stigma and unpleasant smell of cigarettes facilitated smoking cessation, while unknown long-term harms, appealing flavors, and easy accessibility of e-cigarettes hindered vaping cessation. Our study extends previous research, which did not address dual tobacco cessation per se, by providing insights into young adults’ preferences and needs specific to dual tobacco product cessation. These findings inform future interventions for dual tobacco cessation among young adults. Our findings on the types of quitting support that young adults perceived as helpful are similar to previous research on smoking cessation [7] and emerging research on vaping cessation [6]. Young adults using both cigarettes and e-cigarettes may have greater overall nicotine consumption and may need additional education and support for NRT, such as discussing how NRT’s abuse liability compares with cigarettes and e-cigarettes and supports cessation. Intervention content, that our participants expressed would be helpful, included having a quit plan and goals, monitoring quitting progress, getting quitting incentives (eg, health or financial benefits), and learning coping strategies for withdrawal and mental health symptoms. These similarities suggest that frameworks based on existing interventions for smoking cessation and vaping cessation could be adapted to address dual tobacco cessation.

### Targets for Future Interventions

Future dual tobacco cessation interventions should provide sequential cessation support to quitting 1 product prioritized by the individual while cutting down the other product with the explicit goal of quitting both. While some cessation strategies (eg, goal setting, action planning, avoidance of cues, rewards, self-monitoring of behavior, addressing mental health, and peer support) are included in existing cessation programs to address quitting tobacco and nicotine use in general, for dual tobacco cessation, the distinct facilitators and barriers to quitting e-cigarettes (eg, flavors and easy accessibility) and cigarettes (eg, smell) need to be specifically addressed. Importantly, intervention content could emphasize the greater adverse health effects of dual tobacco use compared with single product use and include education on avoiding compensatory effects (ie, switching between tobacco and nicotine products). In addition, specific content discouraging cannabis smoking or vaping as a cessation strategy may be salient for dual tobacco users as co-use of tobacco and cannabis is particularly common among young adults. As our study highlights the heterogeneity of cessation needs and preferences, smartphone apps appear to be a promising channel for delivering personalized support for young adults’ dual tobacco cessation. Dual tobacco cessation interventions may need to account for the fact that people may be at different stages of their quitting journey (eg, quit preparation, quitting period, and relapse prevention) for each of the 2 products. Tailored intervention materials should be iteratively tested and refined with target users, ensuring they equitably address a broad spectrum of cessation needs for diverse audiences.

### Limitations

This study has several limitations. Generalizations from this formative study among a convenience sample of young adults in California are neither possible nor intended. Our findings may reflect unique situations of tobacco use in young adults in California and may be different from other states that have fewer tobacco control policies and higher tobacco use rates than California. Our sample included young adults, with a high proportion of Asian participants reporting dual tobacco use and people interested in quitting vaping and smoking. Thus, findings may not apply to other samples with different characteristics. Our participants were recruited through social media, and thus, they may have been more likely to endorse cessation methods by a smartphone app as smartphones are commonly used for social media among young adults [23]. Although we asked participants about other preferences for dual tobacco cessation, this study focused on the development of a smartphone app for dual tobacco cessation. Given the lack of effective interventions targeting dual tobacco cessation among young adults, future research should explore other communication channels or options for interventions and opportunities to integrate these options into a comprehensive cessation package. Finally, participants’ narratives may be subject to social desirability bias and recall bias.

### Conclusions

This study revealed young adults’ strong desires to quit both e-cigarettes and cigarettes, highlighting the need for interventions targeting dual tobacco cessation. We found a heterogeneity of individual needs and preferences for dual tobacco cessation and suggested key targets for future interventions. Smartphone app–based interventions are promising to offer personalized support for helping young adults quit both e-cigarettes and cigarettes.

## Appendix

Multimedia Appendix 1 Guide for semistructured interviews.

Multimedia Appendix 2 SRQR (Standards for Reporting Qualitative Research) checklist.

## Abbreviations

NRT: nicotine replacement therapy

## Data Availability

Data are not publicly available because the qualitative data, even deidentified, may sufficiently specify the participant and, therefore, cannot be shared. The quantitative data will be shared at a reasonable request to the corresponding author.
