# Supplementary material for: Desires and Needs for Quitting Both e-Cigarettes and Cigarettes Among Young Adults: Formative Qualitative Study Informing the Development of a Smartphone Intervention for Dual Tobacco Cessation
Source: JMIR Form Res. 2024 Oct 22;8:e63156. doi: 10.2196/63156 (PMC11538870; doi:10.2196/63156)
Supplement: Multimedia Appendix 1 [file formative_v8i1e63156_app1.docx]

**Guide for Semistructured Interviews**

**Vaping cessation desire and needs**

- How important is it to you to quit vaping or smoking or both these days?

*Probes:*

- Can you rank the importance of quitting vaping, quitting smoking, and quitting both?
- Why do you think quitting [vaping, smoking, both] the most important for you?
- What are your plans to quit vaping and/or smoking?

*Probes:*

- [Ask only if participants want to quit both] do you want to quit them simultaneously or sequentially? Describe more about your logic for that plan.
- When do you plan to quit [vaping, smoking, both]?
- [Ask only if participants want to either one] How about quitting the other?
- Do you want to have 2 separate interventions for quitting smoking and vaping? Or do you want to have only 1 intervention that integrates support for both?
- What would motivate you or has motivated you in the past to quit smoking and/or vaping?

*Probes:*

- - What kind of quitting support have you experienced in the past?
  - What has helped?
  - What has not been helpful?
  - Did you ever use Quitline or medication to quit?
- What are the barriers to quitting smoking and/or vaping?

*Probes:*

- Have you ever tried to quit smoking, vaping, or both? When did it happen? Why did you not quit successfully?
- What is your biggest barrier?
- What type of supports may help you to overcome those barriers?
- When it comes to quitting smoking or vaping, what do you think would be helpful for you?

*Probes*:

- - Did you ever use Quitline, medication (NRT), self-help guide (leaflet) to quit?
  - How about other supports or help, like app-based, daily text messages, social media, website, hotline, coaching, social support (friend, family)?
  - What are your thoughts about what can be improved or changed which can help people quit smoking?

**Recommendations for smartphone-based intervention**

- What do you think about using a smartphone-based app quit smoking/vaping?
  - What is your ideal app that would help you quit?
- What features make an app more appealing and engaging to young people
- Did you use any apps or smartphone-based programs for quitting before?
  - How were your experiences?
  - Which features if that app/program did you like or not like?
